# From adventures to diagnosis: adolescent behavior in classic fiction through the eyes of newly licensed Czech psychiatrists – a vignette study

**DOI:** 10.3389/fpsyt.2025.1592912

**Published:** 2025-05-23

**Authors:** Vojtech Pisl, Andrea Hodkova, Jiri Hudecek, Marek Pav, Jan Vevera

**Affiliations:** ^1^ Department of Psychiatry, Faculty of Medicine in Pilsen, Charles University, Pilsen, Czechia; ^2^ Department of Psychiatry, Institute for Postgraduate Medical Education, Prague, Czechia; ^3^ Department of Psychiatry, First Faculty of Medicine Charles University in Prague and General University Hospital in Prague, Prague, Czechia; ^4^ Bohnice Psychiatric Hospital, Department of Psychiatry, Praha, Czechia

**Keywords:** diagnostic inflation, cross-cultural comparison, mental disorders, adolescent, psychiatrization of society

## Abstract

**Introduction:**

Youth mental health is a growing concern, with reports of psychiatric diagnoses becoming increasingly prevalent. Among other factors, psychiatrization may inflate the observed prevalence by interpreting experiences previously understood as adversities inherent to human life as symptoms of psychopathology. The current study explores the pathologization of behaviors typical of adolescence by asking contemporary psychiatrists to diagnose and treat a character from a novel who is considered a prototypical teenager of the 19th century: Tom Sawyer.

**Methods:**

A one-page vignette was distributed either in sealed envelopes or via email to 57 psychiatrists who had obtained their license for independent practice between 2021 and 2023 in the Czech Republic. In total, 47 psychiatrists took part, yielding an overall response rate of 82%. The number and frequency of diagnostic conclusions, prescribed medications, and recommended interventions are reported.

**Results:**

Most respondents diagnosed the boy described in the vignette with a psychiatric disorder (94%; CI = 81–98%) and recommended an intervention within the healthcare system (89%; CI =76–96%). Two thirds (62%, CI = 46–75%) recommended pharmacotherapy: antidepressants (27%), antipsychotics (22%), stimulants (13%), and anxiolytics (2%); 68% (53–80%) recommended psychotherapy. Nonmedical interventions (e.g., counseling, social services) were recommended by 49%.

**Conclusions:**

The experiences of an adolescent boy, once interpreted as normative “adventures” in the 19th century, were recognized by newly certified psychiatrists as psychiatric disorder. These findings illustrate the extent of psychiatrization over time and suggest that expert diagnoses may substantially contribute to the overinterpretation of problems of living as psychiatric disorders.

## 1 Background

Estimates suggest that around 13 percent of children and young people aged 19 and under in the EU suffer from a mental health condition (1). Experts warn that youth mental health is deteriorating (2). For instance, the state of child and adolescent mental health was called alarming in Czechia (3) and led to a request to declare national emergency in the US (4). While the prevalence of adolescent psychiatric diagnoses has increased by 40% within the last decade in Czechia, and a steady increase is expected (5), epidemiological data reveal a large treatment gap in addressing young people’s mental health problems (6–8). The steep rise in epidemiological numbers may, however, be partly caused by social and methodological factors affecting the observed prevalences, such as the increased readiness of the general public to perceive the adversities of life as psychiatric disorders; diagnostic inflation may also contribute to the prevalence increases (9–11). These phenomena are part of a wider cultural shift described as psychiatrization: a process through which “psychiatric institutions, knowledge, and practices affect an increasing number of people, shape more and more areas of life, and further psychiatry’s importance in society as a whole” (12).

Psychiatric disorders are formed by societal needs and historical contexts, in addition to biological factors (13, 14). Adverse human traits or experiences can be explained as an inevitable experience inherent to human life, spiritual challenges, or the consequences of personal choices or sociopolitical processes (15). These explanations are, however, gradually being replaced by the biomedical model explaining adversities as psychiatric disorders (15). Indeed, linguistic data show that terms describing emotionally challenging experiences or unwanted mental traits such as “grief”, “sad” or “restless” are increasingly linked to the biomedical model of human suffering (16, 17). Moreover, the concepts of psychopathology such as “anxiety”, “depression” or “trauma” have become broader in meaning in recent decades at least (18–21), expanding the variety of experiences that are interpreted as psychiatric disorders. The concept breadth of psychopathology is related to self-diagnosing and is negatively correlated with age (22, 23), making young people most vulnerable to the adverse effects of psychiatrization.

A conceptual analysis of psychiatrization highlights how the medicalization of everyday adversities – once considered beyond the scope of psychiatry – can “co-produce avoidable patient careers, create dependencies on mental health services, and ultimately promote disempowering changes to subjectivity and sense of self” (12). On a broader societal level, this shift toward psychiatrization may be reshaping human identity, encouraging individuals to perceive themselves as inherently vulnerable rather than resilient in the face of life’s challenges (24, 25). Given how highly exposed young people are to social media saturated with mental health content, they may adopt the lens of psychiatry as the primary explanation for emotionally challenging experiences (26). The consequences may include self-diagnosis that worsens well-being through self-fulfilling prophecies, formal diagnoses that contribute to overtreatment, stigmatization, and the erosion of personal authority over one’s experiences, epistemologies, and identity (11, 27–31).

The mechanisms converting subtle cultural shifts into rising prevalence rates of mental disorders were further described by the Prevalence Inflation Hypothesis (11). It highlights how greater public awareness and reduced stigma around mental health have increased reporting and access to care, especially for milder cases. At the same time, it cautions that these factors, along with diagnostic expansion and sociocultural influences, may inflate prevalence rates without reflecting true increases in the incidence of mental disorders. Moreover, the supply of treatment facilitates demand (26), making acute psychiatry services “The Only Open Door” to adolescents and their parents seeking immediate help in difficult life situations (32).

Because the great majority of studies documenting psychiatrization is based on data from surveys and medical records that are mostly limited to recent decades, we aim to document psychiatrization over a longer period. Therefore, the current study aims to explore the diagnostic and treatment decisions of modern psychiatrists assessing a young person presenting to a psychiatry clinic with an experience that would have been considered healthy two centuries ago. Specifically, we explore what diagnosis and treatment newly certified psychiatrists might assign to a literary character representing the typical, healthy adolescent of the mid-19^th^ century (33–35): Tom Sawyer.

## 2 Methods

### 2.1 Tom Sawyer as the average boy of his time

While Tom Sawyer is a fictional character, his author states that “[m]ost of the adventures recorded in this book really occurred” and that “Tom Sawyer … is a combination of the characteristics of three boys I knew” (35). By linking the novel to his own childhood and revealing his wish to “pleasantly remind adults of what they once were themselves” (ibid), Samuel Clemens (known under his pen name Mark Twain) presents the novel as a description of growing up in the middle of the 19^th^ century. Indeed, *The Adventures of Tom Sawyer* is accepted as a source of information about the 19^th^ century (e.g., 33, 34), being described as “a window into the southern United States of the nineteenth century” (34). While some episodes have obvious fictional aspects, the first chapters describe the realistic struggles of an adolescent unwilling to comply with the requirements of his guardian, finding his routine life too boring, demanding, and unfair.

### 2.2 Materials

A page-long vignette, based on the 3^rd^ chapter of the novel (35), describes a 12-year-old orphan brought by his aunt and guardian for suicidal proclamations following a domestic dispute. According to his guardian, the patient shows signs of behavioral disorders, cheats at school and is disruptive, is reluctant to do household chores, and is popular, yet manipulates his peers. The guardian admits to slapping him in an argument, which led him to run away from the house and only return at about 10pm. The patient himself claims that he was wrongfully accused of breaking a sugar bowl and was punished with such a slap that he fell off his chair. He shouted at his aunt that he would prefer not to exist and went to the river, imagining how he might drown, before calming down and heading back home. Finally, the vignette described the present state of the patient as calm, oriented, and with no current aggressive or suicidal tendencies. The page with the vignette ended with the following questions addressing key decisions that are to be routinely answered by a psychiatrist at the end of an interview with a patient:

1. What diagnosis would you report at the end of your examination?
2. What would be your recommendations?
3. If you would indicate pharmacotherapy, what medication would it be?

These questions are the standard conclusion to a psychiatric examination and do not imply the need for a psychiatric diagnosis and/or medication. Content validity was ensured by two senior psychiatrists and one senior child and adolescent psychiatrist, who reviewed the clinical scenario to confirm its realism, completeness, and clinical relevance. A fourth question, which is not discussed in this study, tapped the reaction to a hypothetical deterioration in the patient’s status in the future was also included. See the vignette and questions in the **Supplementary Materials**.

### 2.3 Sample

The vignette was distributed to 57 Czech psychiatrists who had received their license for independent practice between 2021 and 2023. Of the total sample, 47 responded (response rate 82%).

### 2.4 Licensing psychiatrists in the Czech Republic

Since July 2017, it takes at least 4.5 years after graduating from medical school in the Czech Republic to qualify as a psychiatrist. After 2.5 years, doctors take a core exam, then complete 2 years of their own specialized training. During this time, they must complete mandatory clinical internships, and participate in certified courses and psychotherapy training. The final boarding exam includes a practical exam – a patient examination and a theoretical exam – 2 professional questions and defense of a professional theoretical thesis. After successfully passing the exam, they obtain specialized competence in the field of psychiatry and can practice independently. There are two rounds of boarding exams each year, organized by one of six faculties of medicine, which take turns to administer them.

In 2023, there were 1,361 practicing psychiatrists serving a population of 10.9 million in Czechia. Approximately 30 new psychiatrists pass the board certification exam each year (36).

### 2.5 Procedures

The participants were recruited following their boarding exams. Specifically, all participants who had passed the exam in June 2023 at the Faculty of Medicine in Pilsen, Charles University (28 psychiatrists), or in December 2023 at the 1st Faculty of Medicine in Prague, Charles University (21 psychiatrists), were invited to participate by the corresponding author (JV), who was a member of both boarding committees. The corresponding author informed them that we were anonymously assessing diagnostic consensus among physicians. Almost all of them agreed, with the response rate being 75% (*n* = 21) in Pilsen and 95% (*n* = 20) in Prague.

Those who passed the boarding exam in Pilsen received the case vignette via email, along with a request to complete the questionnaire and informed consent and send it to a third party, ensuring that their identity would remain unknown to any of the study authors. In Prague, immediately after passing the boarding exam, the physicians received an envelope containing the vignette, questionnaire, and informed consent from the corresponding author (JV). They were instructed to leave the sealed envelope and informed consent separately at the secretariat. No control measures were applied during the process – they were not required to submit the envelope and could return it blank or with unanswered questions. These cases were counted as nonrespondents.

To increase the number of respondents, all psychiatrists employed at the largest psychiatric hospital in the Czech Republic, Psychiatric Hospital Bohnice (6 invitations, 4 responses; response rate 67%), or at the University Hospital in Pilsen (2 invitations, 2 responses; response rate 100%) who had passed the boarding exam in 2021 and 2022 were also invited. The procedure was identical to that used in Prague.

Regardless of whether or not they completed the questionnaire, all participants were encouraged to request a free clinical consultation in exchange for their time. No other incentives or rewards were provided to the respondents.

The study was approved by the Ethics Committee of University Hospital Pilsen and the Faculty of Medicine in Pilsen, Charles University (433/23).

### 2.6 Coding and analysis

Respondents’ answers were coded into categories independently by VP and JV, with differences resolved by agreement. Diagnoses were coded according to the ICD-10, which is currently used in Czechia. Only the primary diagnoses that would be assigned during the initial examination were coded; any further differential or potential diagnoses that may be considered during more extensive assessment were ignored. One respondent expressed the need for more information before deciding whether to give *any* diagnosis and was coded as not assigning a diagnosis. The recommended interventions were coded as counseling & social interventions (e.g., educational interventions, psychoeducation, social and legal protection services), psychotherapy (family therapy, CBT, psychotherapy, etc.), outpatient psychiatric care, and hospitalization. When multiple interventions were recommended, all of them were coded. The recommendation of an outpatient diagnostic appointment (e.g., psychological or neurological assessment) was ignored for not being an intervention; psychiatric hospitalization was considered irrespective of its diagnostic or therapeutic purpose. When the respondent described more possible scenarios, the least restrictive recommendation was coded (e.g., for “hospitalize in case of further emotional lability, otherwise psychotherapy” psychotherapy, but not hospitalization was counted). The results are reported as proportions with 95% confidence intervals calculated in *R* version 4.3.0 (37).

## 3 Results

Of 47 respondents, 44 (94%, CI = 81–98%) diagnosed the fictional patient with a psychiatric disorder. Specifically, 70% (51–79%) of the respondents diagnosed behavioral and emotional disorders in childhood, 15% (7–29%) a stress or adjustment disorder, 6% (2–19%) anxiety-depressive or depressive disorder and 4% (1–16%) a personality disorder, while three respondents (6%; CI = 2–19%) reported no diagnosis (**Table 1**).

**Table 1:** Interventions indicated by nosological entity diagnosed.

Recommendations / diagnoses		Total recommendations		Stress / adjustment disorder (F43)		Hyperkinetic disorder (F90)		Conduct disorder (F91)		Mixed disorders of conduct emotions (F92)		Other (2 x F32, 2 x F41, 1 x F60)		No diagnosis	
**Total diagnoses**		**47**	**100%**	**7**	**14.89%**	**6**	**12.77%**	**20**	**42.55%**	**6**	**12.77%**	**5**	**10.64%**	**3**	**6.38%**
**Counseling / social services**		**23**	**48.94%**	4	17.39%	5	21.74%	9	39.13%	1	4.35%	1	4.35%	3	13.04%
**Outpatient psychotherapy**		**32**	**68.09%**	3	9.38%	4	12.50%	16	50.00%	3	9.38%	4	12.50%	2	6.25%
**Outpatient psychiatry**		**7**	**14.89%**	3	42.86%	0	0.00%	2	28.57%	2	28.57%	0	0.00%	0	0.00%
**Psychiatric hospitalization**		**8**	**17.02%**	0	0.00%	2	25.00%	4	50.00%	2	25.00%	0	0.00%	0	0.00%
**Pharmacotherapy**		**29**	**61.70%**	2	6.90%	6	20.69%	13	44.83%	5	17.24%	3	10.34%	0	0.00%
Of which*	Antipsychotics **,***	**11**	**23.40%**	1	9.09%	1	9.09%	9	81.82%	0	0.00%	0	0.00%	0	0.00%
	Antidepressants *,**	**13**	**27.66%**	1	7.69%	0	0.00%	4	30.77%	5	38.46%	3	23.08%	0	0.00%
	Stimulants *	**6**	**12.77%**	0	0.00%	5	83.33%	1	16.67%	0	0.00%	0	0.00%	0	0.00%
	**Anxiolytics**	**1**	**2.13%**	0	0.00%	0	0.00%	1	100.00%	0	0.00%	0	0.00%	0	0.00%

*One respondent recommended a combination of antidepressants and stimulants. **One respondent considered either antipsychotics or antidepressants. ***One respondent considered mood stabilizers as a possible alternative to antipsychotics; this is not displayed in the table. Summaries printed in bold.

Two-thirds of the respondents (62%, CI = 46–75%) recommended pharmacotherapy. Antidepressant medication was the most common prescription (27%; CI = 15–42%; often SSRIs, particularly fluoxetine), followed by antipsychotics (22%; CI = 12–37%; particularly Risperidone), stimulants (13%; CI = 5–26%); and anxiolytics (2%; one respondent); one respondent also considered mood stabilizers as an alternative to antipsychotics. In addition to medication, 17% (8–31%) of the respondents recommended hospitalization, 17% (8–31%) recommended outpatient psychiatric care, and 68% (53–80%) recommended psychotherapy. Altogether, an intervention within the healthcare system was recommended by 89% of the respondents (76–96%; excl. counseling) or 70% (55–82% excl. psychotherapy and counseling), respectively ^1^. Interventions outside of the healthcare system, such as counseling or social services activization, were recommended by 49% (34–64%) of the respondents.

## 4 Discussion

Our results reveal that if Tom Sawyer – a boy considered healthy in the 19th century – was to consult a modern, newly certified psychiatrist, he would likely be diagnosed and treated. These findings show an increase in the willingness to classify adolescents’ mental struggles as psychopathologies since the 19^th^ century. Moreover, they imply that overinterpretation of life problems as psychiatric disorders described by the prevalence inflation hypothesis (11) may be facilitated by medical diagnoses, in addition to the effects of self-diagnoses. Our observation is consistent with previous findings of concept creep (18, 19, 21) and diagnostic inflation (14, 27, 38, 39), and illustrates the extent to which the concept of psychopathology has broadened over the course of two centuries.

The reasons why the majority of newly certified psychiatrists assigned a psychiatric diagnosis and pharmacotherapy to the adolescent described in our vignette remain uncertain, but several plausible explanations can be considered. Because the current diagnostic practice focuses on avoiding false negatives (i.e., failing to recognize a disorder) at the expense of increased false positive rates (38), it is likely that the individuals presenting to a psychiatric service may receive a diagnosis, whether or not they are mentally disordered. Furthermore, a psychiatrist on duty may recommend—but cannot directly provide—interventions aimed at promoting well-being outside the healthcare system, such as outdoor adventure programs, initiatives to enhance social inclusion, or increased access to nature-based activities (40–43). As a result, medication may often be used to address issues that could be more appropriately managed through non-pharmacological means. This trend may be especially pronounced in younger psychiatrists, because psychiatrization was negatively correlated with age in samples of general population (22).

These results do not imply that the case formulation of Tom Sawyer as a psychiatric patient was not justified. On the one hand, it may be seen as an act of defensive medicine: providing interventions as a safeguard against the possibility of being blamed for not doing so. On the other hand, interpreting Tom’s behavior as normal in the 1850s may have resulted from underdiagnosis in the 1850s. Finally, Tom’s behavior may have been normal in the 19^th^ century and indicative of psychopathology today due to a cultural shift in the meaning of his experiences.

Underdiagnosis and overdiagnosis are related but independent problems. While non-disorders are sometimes treated as mental health conditions, many clinically significant conditions remain untreated (10). Despite a steep rise in the use of mental health treatments over recent decades, population-level mental health has not improved (44, 45). In parallel, anxiety and depression are the most prevalent disorders among adolescents, but most young people affected do not receive professional care (46–48), although mental health interventions are more likely to be effective if delivered during adolescence (49).

The costs and benefits of diagnosing and treating the protagonist of a novel can only be conjectured. An orphan struggling with the perils of adolescence may benefit from mental healthcare. The medication prescribed may be effective in suppressing the problems reported by Tom and his aunt (50, 51). Psychotherapy often has positive effects on these problems, well-being, and brain development (52, 53). However, these undoubted benefits may be counterbalanced by at least four substantial downsides. First, psychiatric diagnoses can reinforce a view that problems stem from an internal defect, leading to passivity and shaping identity through self-labeling and stigma (28, 31, 54, 55). Second, medicalizing Tom’s behavior shifts attention away from social and educational contexts, limiting systemic interventions and overburdening child psychiatry with less severe cases (27, 31, 56, 57). Third, pharmacotherapy carries known risks, especially in developing brains; antidepressants affect the microbiome (58, 59), and antipsychotics—widely prescribed beyond psychotic disorders—have significant side effects (60–65). Fourth, the psychiatrization of human hardships characteristic of adolescence may deter culturally embedded, “local” coping strategies and meaning-making that foster resilience (66).

### 4.1 Limitations

In addition to the obvious limitations of the sample of newly licensed Czech psychiatrists, the data may be affected by the evaluation of a vignette instead of a physically present patient. Firstly, the clinicians may have turned to heuristics and presumptions to complete the missing information that would be apparent with a real patient. Evaluating a vignette for research purposes may have led to a more careful examination than a routine check of a patient, perhaps biasing the chances of questioning whether the patient may need psychiatric care in the first place. At the same time, the absence of opportunity to experience the patient’s behavior in person may have led to the selection of the most likely option given the limited amount of information available. Secondly, the respondents were directly asked about a diagnosis to be reported rather than asking whether the case study represents a psychiatric patient or not. This design mimics the clinical reality codetermined by IT systems requesting a diagnosis to be completed in patients’ records, but assigning a diagnosis may be affected by compliance with the survey question. Similarly, the direct question about medication may have biased the respondents toward pharmaceutical options, despite asking conditionally “which medication … *if indicated*”. Finally, unlike the “real” Tom Sawyer, the patient in our vignette was brought to a psychiatry clinic for examination. The fact that a child is presented to a psychiatrist by his guardian may serve as a source of information for the clinician. By providing a diagnosis and/or treatment, the clinicians may have reacted to an implicit request from the patient’s family, expressed by turning to healthcare services. In general, the data collection arrangements may have prompted the medical solutions to the given case, perhaps tempting the respondents away from the search for nonmedical solutions and interventions. However, these factors do not differ from the pressures and motivations present during routine clinical assessment.

### 4.2 Implications

Clinical practitioners should be sensitive to the side effects of pharmacotherapy as well as patients’ subtle cognitive reactions to knowing that they are being diagnosed and treated, particularly when approaching patients with minor manifestations of symptoms. From a research perspective, the interactions between objective human conditions and cultural notions of normalcy and psychopathology should be studied, because both codetermine the experiential qualities of mental suffering. Educational and promotional campaigns should counterbalance the increasing awareness of which experiences may constitute psychopathology with equally important information about the plethora of experiences likely to be normative.

### 4.3 Conclusions

The experiences of an adolescent boy that were described as common adventures in the 19^th^ century were interpreted as symptoms of a psychiatric disorder by modern psychiatrists. The consequences of such a shift in interpretation may include the adverse effects of medication and stigma resulting from the diagnosis, failure to provide alternative interventions, and the dissemination of resilience-eroding narratives whose effectiveness has not been researched or proven. Moreover, such psychiatrization may give rise to the spurious or inflated notion of a mental health epidemic with the potential to become self-fulfilling.

## Data Availability

The raw data supporting the conclusions of this article will be made available by the authors, without undue reservation.
