# Supplementary material for: From adventures to diagnosis: adolescent behavior in classic fiction through the eyes of newly licensed Czech psychiatrists – a vignette study
Source: Front Psychiatry. 2025 May 23;16:1592912. doi: 10.3389/fpsyt.2025.1592912 (PMC12143267; doi:10.3389/fpsyt.2025.1592912)
Supplement: Supplementary file 1 [file DataSheet1.docx]

## Vignette and questions

A 12-year-old boy was brought in by a guardian for acute psychiatric evaluation due to suicidal proclamations in response to a domestic dispute.

In guardianship of his aunt (mother's sister). Both parents died when the patient was young, he does not know the circumstances, he does not remember them. He lives in his aunt's house along with a cousin of the same age. Their economic situation is satisfactory. They live in their own house without a mortgage. The patient is physically healthy, with no history of serious illness. He denies alcohol and drugs.

Current illness: According to the guardian, behavioral disorders are present in the long-term, the patient is disobedient and lies. He often breaks and destroys things, albeit rather unintentionally. Reluctant with chores, does them only after repeated reminders. Quite popular with peers, member of an informal group of friends, but there are frequent complaints about his behavior, particularly at school. He and his friends are often disruptive in class and are disciplined. Often fights with other boys. The boy does not like to study, he tries to avoid school duties and often looks for all kinds of excuses not to fulfill them. He cheats at school, falsifies grades (improving them). Sometimes he is absent from school without permission. The guardian also mentions that the boy often manipulates his peers, using them for his own benefit.

Today, there was a conflict between the aunt and the patient. She believed he had broken a valuable sugar-bowl and she admits to hitting the boy. She slapped him. In response, there was an argument: the patient felt hurt by the physical punishment. He shouted that it would be better not to exist, that he wanted to get deathly ill to get rest. He ran away from the house. The aunt was worried about him, they were looking for him. He came home by himself around 10 p.m.

According to the patient: He got angry when his aunt wrongly accused him of breaking something he didn't break. He didn't do it. He was slapped so hard that he fell off his chair. They shouted at each other. He said it would be better not to be. He ran out to the river, where he was imagining how he could drown. Gradually, he calmed down, the thoughts then receded, and he returned home alone. But at that moment he meant it. He doesn't like school, it's boring. He would prefer to spend time with his friends.

Current status: currently calm, vigil, lucid, fully oriented, psychomotor speed normal, he approaches the interview relatively willingly, contact is valuable, answers logically, closely, phoria situationally decreased, emotionality currently unstable, tendency to tearfulness, slightly anxious reactivity, intellect roughly normal, cognitive and mnestic functions approximately without disorders, currently without hyperactivity and impulsivity, but described in the home and school environment, behavioral disorders present, currently without auto- or hetero-aggression, suicidal proclamations recently in response to a conflict in the home environment, now he denies them. Undisturbed sleep. No eating disorder.

*How would you proceed if this patient was brought to your service in a psychiatric hospital or to your ambulance?*

*l. What diagnosis would you report at the end of your examination?*

*2. What would be your recommendations?*

*3. If you would indicate pharmacotherapy, what medication would it be?*

*4. If you eventually had hospitalized the patient and for the second and third day of the treatment, apathy, anhedonia, absence of thought and perception disorders, non-compliance with the ward regime, conflicts with fellow patients, attempts to escape from the ward, suicidal ideation would persist, what medication would be probably prescribed?*
